# Correlation analysis of adverse outcomes for the selective reduction of twin pregnancies

**DOI:** 10.1186/s12884-022-04754-4

**Published:** 2022-05-18

**Authors:** Li Gao, Qian-Qian Xu, Shan Wang, Yuan-Qing Xia, Xin-Rong Zhao, Yi Wu, Ren-Yi Hua, Jin-Ling Sun, Yan-Lin Wang

**Affiliations:** 1 Division of Maternal-Fetal Fetal Medicine, Prenatal Diagnosis Center, The International Peace Maternity and Child Health Hospital, School of Medicine, Shanghai Jiao Tong University, Shanghai, China; 2 Shanghai Key Laboratory of Embryo Original Diseases, Shanghai, China; 3 Shanghai Municipal Key Clinical Specialty, Shanghai, China

**Keywords:** Transabdominal, Selective reduction, Twin pregnancy, Adverse outcomes

## Abstract

**Background:**

Due to the extensive development of assisted reproductive technology, the number of twin pregnancies has increased significantly over recent decades. Twin pregnancy is the most representative type of multiple pregnancies and is associated with high infant morbidity and mortality. Perinatal complications of twin pregnancy are also markedly increased compared with those of single pregnancy. Transabdominal selective reduction (SR) is a remedial intervention. This study aimed to research the adverse outcomes of transabdominal selective reduction of twin pregnancy and the correlation between the reduction week and pregnancy outcomes.

**Objective:**

The purpose of this study was to examine the adverse outcomes of the transabdominal selective reduction of twin pregnancy and the correlation between the reduction week and pregnancy outcomes.

**Methods:**

A retrospective cohort study of the transabdominal reduction of twin pregnancy was conducted in a single prenatal diagnosis medical centre from September 2012 to October 2020. According to chorionicity, women with twin pregnancies were divided into 2 groups: dichorionic (DC) twin pregnancies and monochorionic (MC) twin pregnancies. Women with DC twin pregnancies underwent potassium chloride reduction, and those with MC twin pregnancies underwent radiofrequency ablation (RFA). The reduction indications included pregnancy complications, foetal abnormalities, and maternal factors. The perinatal outcomes of different chorionic twins after reduction were analysed. Each foetus with an adverse outcome was included. The relative relationship between the reduction weeks and delivery weeks of twins was examined by correlation analysis.

**Results:**

A total of 161 women were included in this study. A total of 112 women had DC twin pregnancies, and 49 women had MC twin pregnancies. Preterm delivery rates were significantly higher in the MC twin reduction group than in the DC twin reduction group prior to 37 weeks (53.1% vs. 29.5%, *P* = 0.004). The mean gestational age at delivery of the foetuses in the DC twin group that underwent SR was significantly older than that of those in the MC twin group that underwent SR (36.9 ± 4.0 vs. 33.5 ± 6.6 weeks, *P* = 0.001). The number of DC twins that underwent SR and were delivered after 37 weeks was obviously greater than that of the MC twins that underwent SR (70.5% vs. 46.9%, *P* = 0.004). The foetal survival rate was 95.5% in the DC twin reduction group and 77.6% in the MC twin reduction group. If the indication of TTTS was not included, there was no significant difference in the foetal survival rate of the DC and MC twin reduction groups (95.5% vs. 86.2%, *P* = 0.160). Cotwin death 1 week after reduction was greater in the MC group (6.1% vs. 0%, *P* = 0.027). Compared to other indications, this finding indicated that a significantly lower proportion of women remained undelivered after selective reduction with the indication of TTTS. There was a significant negative correlation between the reduction weeks and delivery weeks of the two groups (*P* < 0.01), and the best opportunity for reduction was before 22 weeks of gestation.

**Conclusion:**

These findings highlighted an obviously negative correlation between the reduction week and delivery week. The transabdominal selective reduction of twin pregnancy should be considered for a lower rate of miscarriage or premature delivery if the reduction week takes place earlier in pregnancy. The rate of preterm delivery was the lowest when transabdominal selective reduction was completed before 22 weeks of gestation. Compared with other RFA indications, a higher rate of premature delivery was shown for MC twins with a reduction indication of TTTS. TTTS with sIUGR might be one of the reasons for the adverse outcomes of reduction for MC twin pregnancy.

## Introduction

The rate of multiple pregnancies, especially that of twin pregnancies, has increased rapidly since assisted reproduction technologies (ARTs) have become almost explosively common worldwide in the past few decades [1, 2]. Multiple pregnancies are related to higher risks of adverse pregnancy outcomes than singleton pregnancies [3, 4]. The incidence of spontaneous abortion, prematurity, foetal dysplasia and maternal complications rise along with the foetal number [5]. Compared with singleton pregnancies, multifoetal pregnancies are associated with an approximately fivefold increased risk of stillbirth and a sevenfold increased risk of neonatal death. These adverse pregnancy outcomes are mainly due to complications of prematurity, and the risk of adverse outcomes can be lowered by reducing the number of foetuses [6, 7]. MC twin pregnancies are associated with specific complications due to placental sharing, including a higher risk of twin-twin transfusion syndrome (TTTS), foetal growth restriction, early preterm delivery and perinatal mortality. When one of the MC twins dies, with placental vascular circulation, cerebral injury or demise of the normal cotwin may occur [8]. The SR of a monochorionic (MC) twin, which is discordant for either serious structural anomalies or severe growth restriction, can, in some cases, improve the outcome for its cotwin [9, 10].

To date, few studies have analysed the correlation between reduction indications and the outcomes of MC twins. Few studies have explored the optimal choice for reducing gestational age for twins. The purpose of this study was to research the perinatal complications of transabdominal SR of twin pregnancies and the correlation between the reduction week and pregnancy outcomes.

## Materials and methods

### Materials

The research examined the data of all women with twin pregnancies who underwent reduction at the International Peace Maternity & Child Health Hospital (IPMCHH), Shanghai Jiaotong University, from September 20, 2012, to October 9, 2020. This study was approved by the Ethics Committee of the hospital (No. GKLW 2015–62). According to chorionicity, the twin pregnancies were divided into 2 groups: dichorionic (DC) twin pregnancies and monochorionic (MC) twin pregnancies. Women with DC twin pregnancies underwent potassium chloride reduction, and those with MC twin pregnancies underwent radiofrequency ablation (RFA).

Indications for DC twin reduction included foetal factors (such as chromosomal abnormalities and foetal abnormalities) and maternal factors (such as cervical insufficiency). Indications for the use of RFA for women with MC twin pregnancies at our centre include TRAP sequence, obligate lethal discordant anomalies, severe TTTS with proximate placental cord insertion sites or sFGR type II with abnormal ultrasound blood flow indicators.

The perinatal outcomes of different chorionic twins after reduction were analysed. Each foetus with an adverse outcome was included. The relative relationship between the reduction weeks and delivery weeks of the twins was examined by correlation analysis. According to chorionicity, the perinatal outcomes of the twins after SR were analysed.

### Statistical analysis

All statistical analyses were performed by SPSS software (version 21; IBM Corp). Descriptive statistics included numbers and percentages for categorical variables. The data for delivery weeks are presented as the mean (±SD). Maternal age and prepregnancy BMI were assessed with the independent-samples t test. χ2 tests were used for the comparison of categorical variables and to generate the relative risks (RRs) for dichotomous outcomes and the mean differences (MDs) for continuous outcomes with 95% confidence intervals (CIs). Incidences were reported with significance accepted at a *p* value< 0.05. Kaplan–Meier survival analysis was performed to compare the proportion of MC twin delivery weeks for different reduction indications. Correlation analysis and ROC curve analysis was used to study the relationship between the reduction week and delivery week.

## Results

### Characteristics of the study population

A total of 161 women with twin pregnancies were included in this study. A total of 112 women had dichorionic (DC) twin pregnancies, and 49 women had monochorionic (MC) twin pregnancies. Women with DC twin pregnancies underwent potassium chloride reduction, and those with MC twin pregnancies underwent radiofrequency ablation (RFA).

The baseline and obstetric characteristics of the study subjects are shown in Table 1. Comparisons between the two groups showed that the delivery age of foetuses in the DC twin group was significantly older than that of those in the MC twin group (*P* < 0.001). The spontaneous pregnancy rate was obviously lower in the DC twin group (24.1% vs. 85.7%, *P* < 0.001), and the rate of in vitro fertilization, as recognized, was clearly higher in the DC twin group (67.9% vs. 8.2%, *P* < 0.001). The proportion of parity equal to 2 was higher in the MC twin group (6.1% vs. 0%, *P* = 0.027).

**Table 1:** Baseline and obstetric characteristics of the study subjects. (According to chorionicity^a^)

Groups	DC	MC	*P*
Total number	112	49	
Maternal age, n (%)			
≥ 40 y	7 (6.3)	1 (2.0)	0.000
35–39 y	23 (20.5)	5 (10.2)	
30–34 y	53 (47.3)	20 (40.8)	
25–29 y	28 (25.0)	15 (30.6)	
20–24 y	1 (0.9)	7 (14.3)	
< 20 y	0 (0.0)	1 (2.0)	
Prepregnancy BMI^a^, n (%)			
≥ 30 kg/m^2^	1 (0.9)	1 (2.0)	0.216
25–29 kg/m^2^	17 (15.2)	8 (16.3)	
20–24 kg/m^2^	65 (58.0)	32 (65.3)	
< 20 kg/m^2^	29 (25.9)	8 (16.3)	
Mode of conception, n (%)			
Spontaneous	27 (24.1)	42 (85.7)	0.000
In vitro fertilization and embryo transfer	76 (67.9)	4 (8.2)	0.000
Superovulation+Artificial Insemination	9 (8.0)	3 (6.1)	0.921
Gravidity, n (%)			
1	47 (42.0)	19 (38.8)	0.705
2	33 (29.5)	15 (30.6)	0.884
3	18 (16.1)	9 (18.4)	0.720
4	6 (5.4)	5 (10.2)	0.434
5	6 (5.4)	1 (2.0)	0.596
6	2 (1,8)	0 (0.0)	1.000
Parity, n (%)			
0	85 (75.9)	30 (61.2)	0.058
1	27 (24.1)	16 (32.7)	0.259
2	0 (0.0)	3 (6.1)	0.027

Chorionicity: Twins were divided into double chorionic twins and mono chorionic twins according to their chorionicity. *DC* Dichorionic Twins, *MC* Monochorionic Twins. *BMI* Body Mass Index, Weight (kg) divided by the square of height(m)

### Obstetric outcomes of SR according to chorionicity

The obstetric outcomes and delivery information of women who underwent SR according to chorionicity are presented in Table 2. The preterm delivery rates were significantly higher in the MC twin reduction group than in the DC twin reduction group prior to 37 weeks (53.1% vs. 29.5%, *P* = 0.004). The mean gestational age at delivery of foetuses in the DC twin group that underwent SR was significantly later than that of the MC twin group that underwent SR (36.9 ± 4.0 vs. 33.5 ± 6.6 weeks, *P* = 0.001). The number of DC twins who underwent SR and were delivered after 37 weeks was obviously greater than that of MC twins who underwent SR (70.5% vs. 46.9%, *P* = 0.004). The foetal survival rate was 95.5% in the DC twin reduction group and 77.6% in the MC twin reduction group. If the indication of TTTS was not included, there was no significant difference in the foetal survival rate of the DC and MC twin reduction groups (95.5% vs. 86.2%, *P* = 0.160). Cotwin death 1 week after reduction was greater in the MC group (6.1% vs. 0%, *P* = 0.027).

**Table 2:** The obstetric outcomes after reduction according to chorionicity

Groups	DC N(%)	MC N(%)	*P*	Risk ratio	95% CI
Total number	112	49	/	/	/
PPROM^a^	3 (2.7)	1 (2.0)	1.000	1.321	0.134–13.026
Preterm Labour	33 (29.5)	26 (53.1)	0.004	0.370	0.185–0.739
Mean GA at delivery (weeks)	36.9 ± 4.0	33.5 ± 6.6	0.001	/	1.379–5.418
GA at birth 24–27 6/7	2 (1.8)	4 (8.2)	0.130	0.205	0.036–1.157
28–31 6/7 weeks	6 (5.4)	5 (10.2)	0.434	0.498	0.144–1.717
32–33 6/7 weeks	2 (1.8)	4 (8.2)	0.130	0.205	0.036–1.157
34–36 6/7 weeks	20 (17.9)	6 (12.2)	0.373	1.558	0.584–4.158
≥37 weeks	79 (70.5)	23 (46.9)	0.004	2.706	1.354–5.410
Foetal survival rate	107 (95.5)	38 (77.6)	0.001	6.195	2.021–18.987
Foetal survival rate (excluding TTTS)^b^	107 (95.5)	25/29 (86.2)	0.160	3.424	0.857–13.678
SGA^c^	5 (4.5)	5 (10.2)	0.301	0.411	0.113–1.491
Perinatal mortality	2 (1.8)	4 (8.2)	0.130	0.205	0.036–1.157
Cotwin death ≤1 week after reduction	0 (0.0)	1 (2.0)	0.304	1.021	0.980–1.063
Cotwin death > 1 week after reduction	0 (0.0)	3 (6.1)	0.027	1.065	0.992–1.144
GDM^c^	16 (14.3)	5 (10.2)	0.479	1.467	0.505–4.258
HDP^c^	8 (7.1)	4 (8.2)	1.000	0.865	0.248–3.021

^a^ *PPROM* Preterm Premature Rupture Of Membranes

^b^ Foetal survival rate (excluding TTTS): The indication of TTTS was not included for women with MC pregnancies who underwent reduction

^c^ *SGA* Small for Gestational Age infant; also known as intrauterine growth retardation; a newborn whose birth weight is below the 10th percentile or 2 standard deviations below the average weight for gestational age. *GDM* Gestational Diabetes Mellitus. *HDP* Hypertensive Disorders of Pregnancy

### Obstetric outcomes of SR according to chorionicity

Table 3 demonstrates 17 foetuses of adverse outcomes. Eleven cases occurred for MC twins, and 6 cases occurred for DC twins. The proportion of spontaneous conception was significantly higher than that of assisted reproduction. TTTS with sIUGR was the main indication for the adverse outcomes of reduction for MC twin pregnancy. The gestational age at reduction was between 16 and 24 weeks of gestation. The main outcome of DC twin reduction was spontaneous abortion, and one case of induced labour occurred because of retained foetal malformation. The outcomes of MC twin reduction included 5 cases of cotwin death, 4 cases of spontaneous abortion, and 2 cases of induced labour for cotwin malformation.

**Table 3:** Cases of adverse outcomes

Case	Chorionicity^b^	G&P^a^	Method of conception	Reduction Indication	Reduction Method	Reduction Week	Delivery Week	Outcome
1	MCDA^b^	G1P0	Spontaneous	foetal anomalies	RFA^b^	17	23	abortion
2	DCDA^b^	G2P0	Superovulation	foetal anomalies	KCL^b^	19	26	abortion
3	DCDA	G2P0	IVF	foetal anomalies	KCL	16	20	abortion
4	MCDA	G2P1	Spontaneous	TTTS, sIUGR	RFA	19	21	stillbirth
5	MCDA	G3P0	Spontaneous	TTTS	RFA	24	27	stillbirth
6	DCDA	G1P0	IVF	foetal anomalies	KCL	23	27	induced labour
7	DCDA	G2P0	IVF	foetal anomalies	KCL	19	20	abortion
8	MCDA	G2P1	Spontaneous	TTTS, sIUGR	RFA	16	16	abortion
9	MCDA	G1P0	Spontaneous	cervical insufficiency	RFA	18	20	abortion
10	MCDA	G1P0	Spontaneous	TTTS, sIUGR	RFA	23	26	abortion
11	MCDA	G1P0	Spontaneous	TTTS, sIUGR	RFA	18	23	induced labour
12	MCDA	G1P0	Spontaneous	sIUGR	RFA	18	22	stillbirth
13	MCMA^b^	G1P0	Spontaneous	TRAPs	RFA	18	26	stillbirth
14	MCDA	G1P0	Spontaneous	TTTS	RFA	24	24	stillbirth
15	MCDA	G2P1	Spontaneous	TRAPs	RFA	16	23	induced labour
16	DCDA	G2P0	IVF	foetal anomalies	KCL	16	29	abortion
17	DCDA	G5P0	IVF	cervical insufficiency	KCL	12	18	abortion

^a^ G&P: Gravidity & Parity

^b^ Chorionicity: Twins were divided into double chorionic twins and monochorionic twins according to their chorionicity. *DCDA* Dichorionic Diamniotic Twins, *MCDA* Monochorionic Diamniotic Twins, *MCMA* Monochorionic Monoamniotic Twins. *RFA* Selective reduction using radiofrequency ablation, *KCL* Selective reduction using potassium chloride

### Comparison of delivery time of MC twins with different indications after reduction

**Fig. 1:**
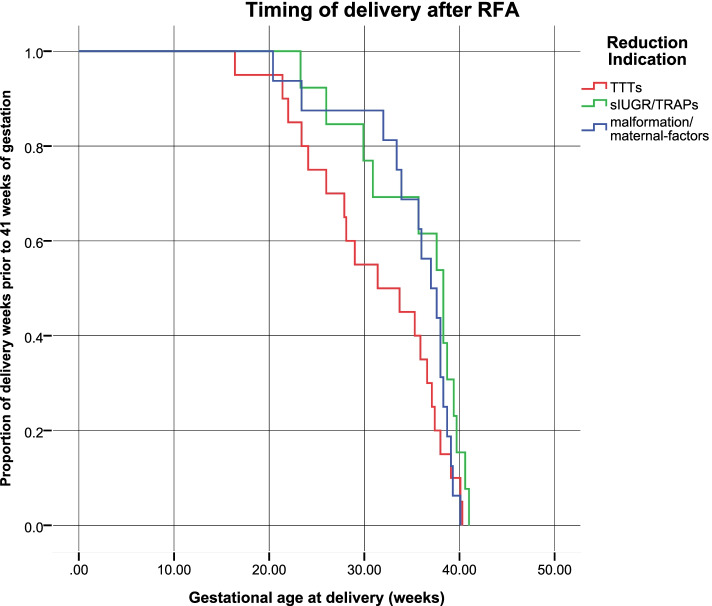
Kaplan–Meier curves according to the three types of indications

Kaplan–Meier curves showed that the proportion of women with complicated MC twin pregnancies remaining undelivered from the time of radiofrequency ablation until 41 weeks’ gestation (Fig. 1). Plots indicated that a significantly lower proportion of women remained undelivered after selective reduction with the indication of TTTS compared to that of women with other indications. An obviously higher rate of premature delivery was shown for women with a reduction indication of TTTS. The rate of premature delivery was similar for other foetal reduction indications for MC twin pregnancies.

### Correlation analysis between the reduction weeks and delivery weeks of twins

**Fig. 2:**
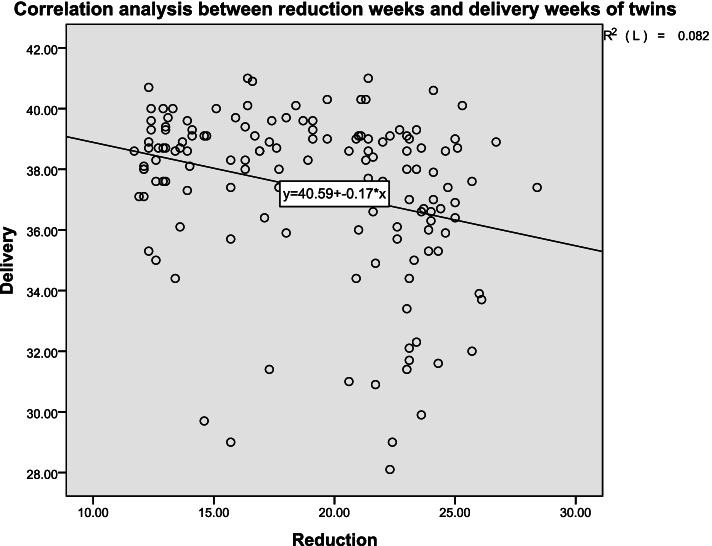
Correlation analysis showed the relationship between the reduction weeks and delivery weeks of 145 foetuses delivered at > 28 weeks’ gestation

Sixteen foetuses with adverse outcomes before 28 weeks of delivery were excluded, and the correlation between the reduction week and delivery week of 145 foetuses was analysed (Fig. 2). The average reduction week was 19.0 ± 4.6, and the average delivery week was 37.3 ± 2.7. The Pearson correlation coefficient was −.287** (*P* < 0.01). There was a significant negative correlation between the reduction week and delivery week.

**Fig. 3:**
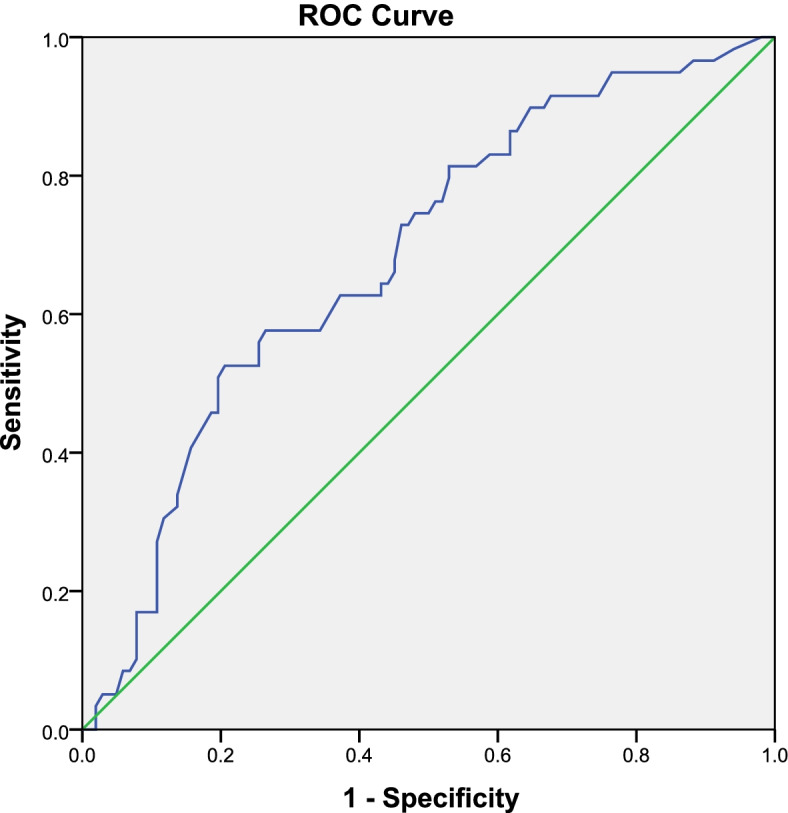
ROC Curve for reduced weeks and delivery gestational age after 37 weeks of all subjects

ROC curves were used to study the relationship between the reduction week and gestational age at delivery of all subjects (Fig. 3). The threshold for gestational age at delivery was set at later than 37 weeks. The area under the curve was 0.683 (*P* < 0.001), and the 95% confidence interval was 0.598–0.767. The Youden Index was 0.319, and the best opportunity for reduction was before 22 weeks of gestation.

## Discussion

Twin pregnancy is associated with an obviously higher risk of preterm birth and adverse pregnancy outcomes than singleton pregnancy. The reduction of a twin pregnancy to a single pregnancy might be performed to decrease the risk due to maternal history, to decrease known complications of twin pregnancy, or for social reasons. Our study showed that the preterm delivery rate was 29.5% in the DC twin reduction group and 53.1% in the MC twin reduction group. The number of DC twins who underwent SR and were delivered after 37 weeks was obviously greater than that of MC twins who underwent SR (70.5% vs. 46.9%, *P* = 0.004). Alvarado [11] et al. found a prematurity rate of 11.8% for preterm delivery at less than 34 weeks in the DC twin reduction group, with a miscarriage rate of 3.6%. This is analogous to our data of a 9% prematurity rate and a 4% miscarriage rate.

We included 17 foetuses with adverse outcomes: 11/49 foetuses (22.4%) were MC twins, and 6/112 foetuses (5.4%) were DC twins. Adverse outcomes included abortion, stillbirth, and cotwin malformation-induced labour. The reduction week for the foetuses was between 16- and 24-weeks’ gestation. TTTS with sIUGR was the main indication for adverse outcomes of the reduction of MC twin pregnancy. The research of Kristi R revealed that sIUGR of the TTTS donor twin was an independent risk factor for donor foetal demise and thirty-day neonatal donor nonsurvival [12]. The coexistence of TTTS and SIUGR was relatively common and suggested the underlying pathophysiological relationship between these two conditions. The cause of TTTS is unequal blood flow, while the cause of sIUGR has been attributed to unequal placental sharing. Different distributions of placental blood flow might be the reason why sIUGR changed the risk of developing TTTS. These insights helped us communicate with the pregnant women and their families before intrauterine treatment.

Our study suggested that RFA is an acceptable method for foetal reduction in complicated monochorionic diamniotic twins, with a foetal survival rate of nearly 80%. The pregnancy success rate following RFA selective reduction was lower for the reduction indication of TTTS (77.6%), but it was higher for other reduction indications (86.2%). There was a dramatic decline in the foetal survival rate of the MC twin reduction group (*P* = 0.001). If the indication of TTTS was not included, there was no significant difference in the foetal survival rate of the DC and MC twin reduction groups (*P* = 0.160).

Our study also indicated that, compared to other indications, a significantly lower proportion of women remained undelivered after selective reduction for the indication of TTTS. A higher rate of premature delivery was shown for women with the reduction indication of TTTS. The rate of premature delivery was similar for other foetal reduction indications for MC twin pregnancies.

RFA for TTTS was associated with worse outcomes than RFA for other indications [13], which might be attributable to the severity of the underlying disease and the poorer cardiovascular statuses of foetuses with TTTS. Hongmei Wang et al. performed 272 RFA procedures for 268 complicated MC pregnancies and recorded the lowest survival rate in the TTTS group (37/64, 57.8%) [14]. This result was similar to our study and previous studies [15, 16]. Kumar [17] reported that sIUGR as an indication for RFA had more favourable perinatal outcomes than other indications. We only proceeded to RFA in cases of moribund TTTS when we felt that antenatal demise of the foetus was inevitable. A case series of TRAP patients undergoing RFA at a single centre reported an overall survival rate of 92% [18]. Several studies reported that the live birth rate of twins after RFA was approximately 70–80% [19–21], which was similar to our research. All procedures were performed by a very small group of experienced foetal medicine specialists in our Foetal Medical Center, ensuring uniformity of the surgical technique and success rate.

According to our research, the average reduction week was 19.0 ± 4.6, and the average delivery week was 37.3 ± 2.7. The Pearson correlation coefficient was −.287** (*P* < 0.01). There was a significant negative correlation between the reduction week and delivery week. ROC curves were used to study the relationship between the reduction week and gestational age at delivery of all subjects in our study. The area under the curve was 0.683 (*P* < 0.001), and the 95% confidence interval was 0.598–0.767. The Youden Index was 0.319, and the best opportunity for reduction was before 22 weeks of gestation. This result suggested that the ultrasonic malformation screening of twins should be performed before 21 weeks of gestation and is conducive for subsequent selective foetal reduction.

Hasson reported that the rate of pregnancy complications (late abortion, preterm delivery) was significantly higher when reduction was carried out after 15 weeks of gestation (36.4% vs. 0%) [22]. Greenberg demonstrated that pregnancy was prolonged for women who underwent reduction in the first trimester (37.3 weeks) compared with the second trimester (36.4 weeks) and third trimester (35.3 weeks) [23]. One study also found that the birth weight of the foetus was significantly increased when SR was performed at a time point that was earlier than 15 weeks [24]. Bigelow et al. suggested that foetal loss and preterm delivery after SR for twin pregnancies was associated with increasing gestational age at the time of the procedure [25]. R. ZEMET’s study [26] aimed to determine the rate of perinatal complications of twin reduction in the late first trimester (11–14 weeks) compared with the second trimester (15–23 weeks) and found that the rates of preterm delivery were significantly higher in women who underwent reduction in the second trimester of pregnancy.

The main strength of our study was the relatively larger sample size, and all procedures were performed by a very small group of experienced foetal medicine specialists. However, there are also several limitations in the study. This was a single-centre study with a relatively small sample size in each subgroup. The neonates in this study were not followed up for long-term neurological development. We will further strengthen our multicentre cooperation and increase the long-term follow-up of newborns.

In conclusion, these findings highlighted an obviously negative correlation between the reduction week and delivery week. Foetal reduction in twin pregnancy should be considered for a lower rate of miscarriage or prematurity if the reduction week takes place earlier in pregnancy. The rate of preterm delivery was the lowest when transabdominal selective reduction was completed before 22 weeks of gestation. This suggested that the ultrasonic malformation screening of twins should be performed before 21 weeks of gestation. Compared with other RFA indications, a higher rate of premature delivery was shown for MC twins with the reduction indication of TTTS. TTTS with sIUGR might be one of the reasons for the adverse outcomes of reduction for MC twin pregnancy.

## Data Availability

The datasets generated and/or analysed during the current study are not publicly available as this is a continuing study but are available from the corresponding author upon reasonable request.
